# Supplementary material for: Metabolite Ratios as Quality Indicators for Pre-Analytical Variation in Serum and EDTA Plasma
Source: Metabolites. 2021 Sep 18;11(9):638. doi: 10.3390/metabo11090638 (PMC8465943; doi:10.3390/metabo11090638)
Supplement: Supplementary file 1 [file metabolites-11-00638-s001.zip › Supplemental Material.pdf]

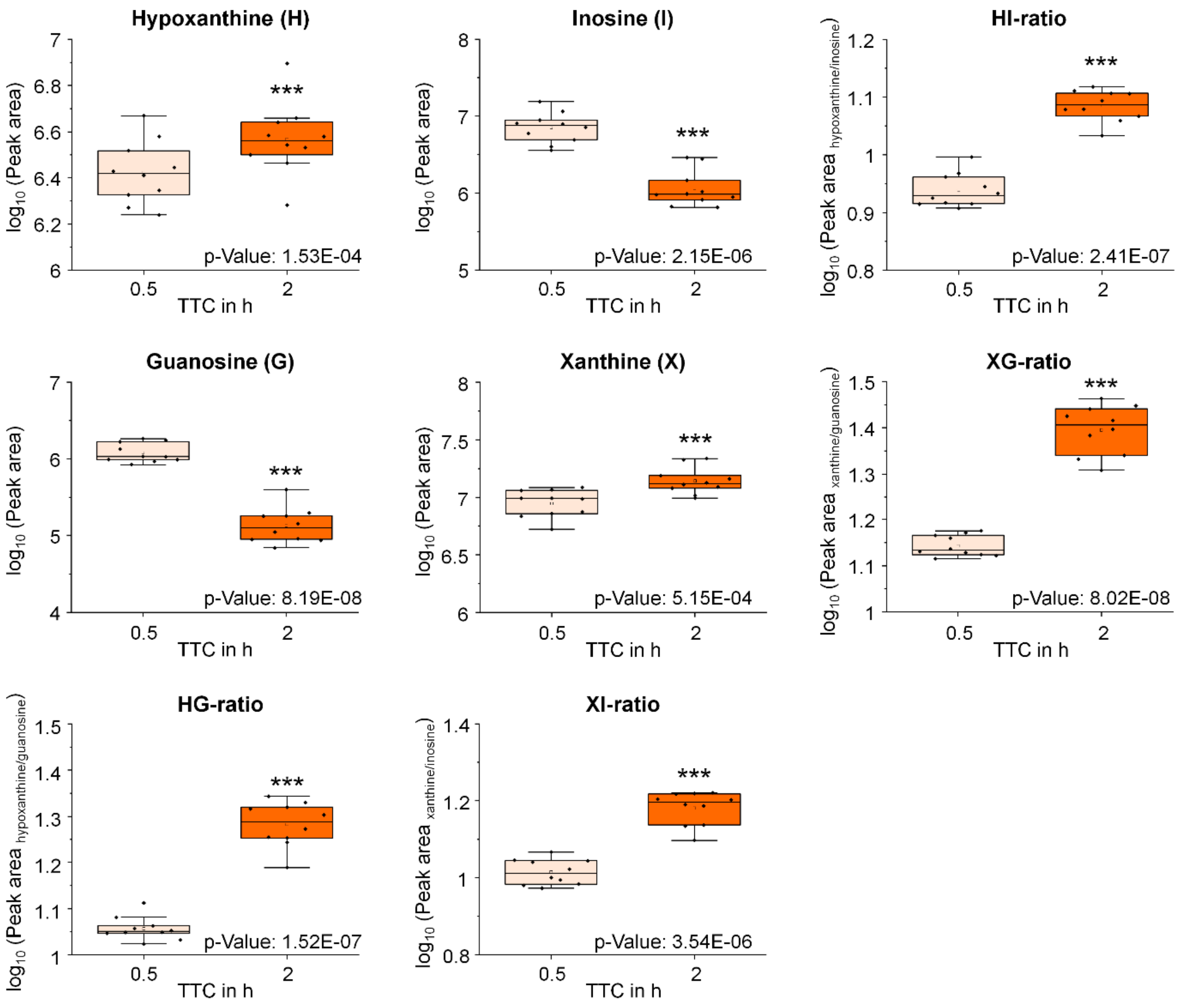

Figure S1: Purine derivatives and their ratios as potential target QIs in human serum

Boxplots visualize the variation of the log_{10}-transformed peak area of the purine metabolites and their ratios in human serum of healthy volunteers after prolonged incubation 0.5 and 2 h at room temperature (*discovery sample 1*, n=10) before centrifugation. Asterisks indicate differences between the time points as indicated by the respective brackets (***p<0.01). P-Values were calculated using paired *t*-tests.

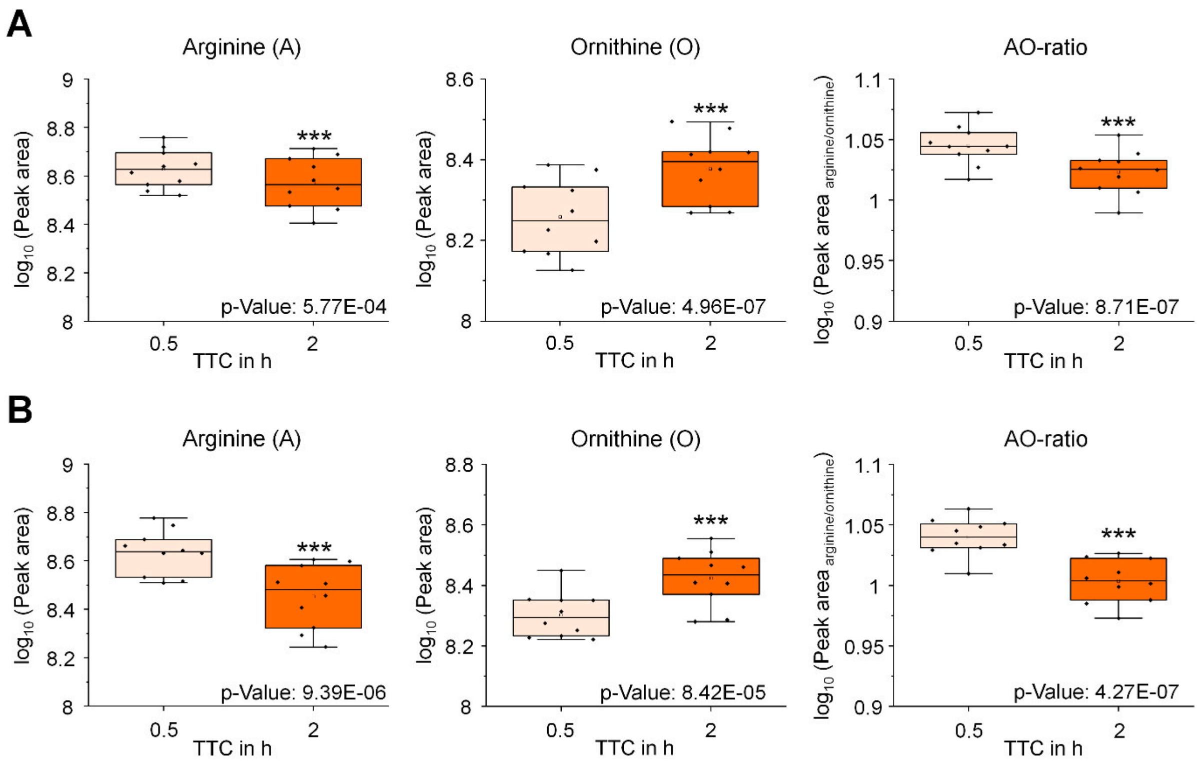

Figure S2: Arginine, ornithine and AO-ratio as potential target QIs in human serum and EDTA plasma

Boxplots visualize the variation of the $\log_{10}$-transformed peak area of the amino acids arginine and ornithine and their ratios in human serum (A) and EDTA plasma (B) of healthy volunteers after incubation at 0.5 and 2 h at room temperature (*discovery sample 1*, n=10) before centrifugation. Asterisks indicate differences between the time points as indicated by the respective brackets (***$p<0.01$). P-Values were calculated using paired *t*-tests.

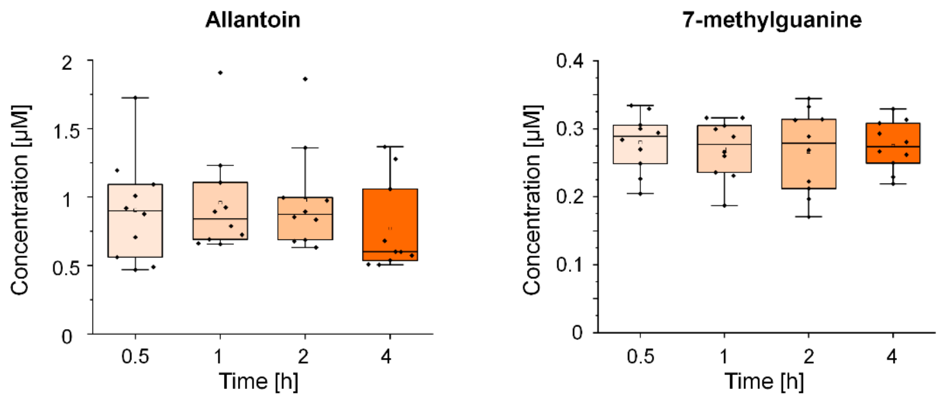

Figure S3: Precentrifugation delay dependent impact on allantoin and 7-methylguanine in serum

Boxplots visualize the variation of the concentration of selected metabolites in human serum of healthy volunteers after prolonged incubation for 0.5, 1, 2 and 4 h at room temperature (*validation sample 1*, n=10) before centrifugation. P-Values were calculated using linear mixed models or paired *t*-tests and corrected for multiple testing using Bonferroni post-hoc test.

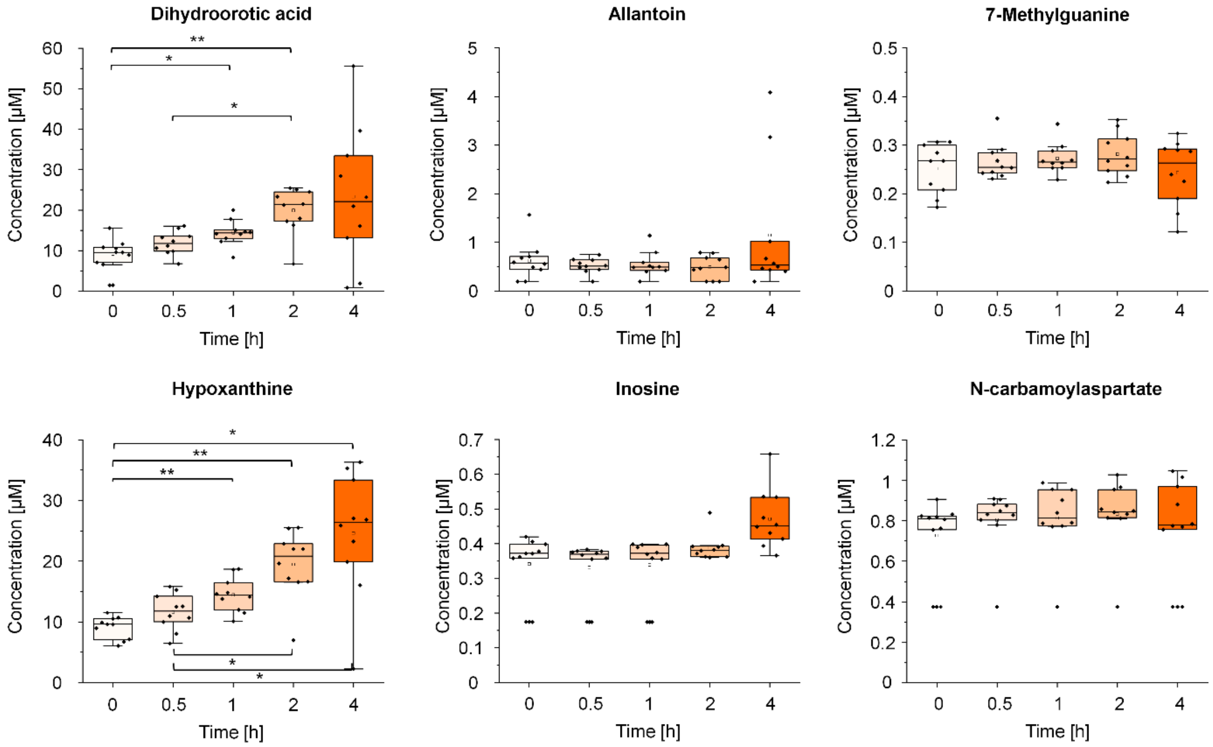

Figure S4: Precentrifugation delay dependent impact on selected metabolites in EDTA plasma

Boxplots visualize the variation of the concentration of selected metabolites in human EDTA plasma of healthy volunteers after prolonged incubation for 0, 0.5, 1, 2 and 4 h at room temperature (*validation sample 1*, n=10) before centrifugation. Asterisks indicate differences between the time points as indicated by the respective brackets (*p<0.05, **p<0.01). P-Values were calculated using linear mixed models or paired *t*-tests and corrected for multiple testing using Bonferroni post-hoc test.

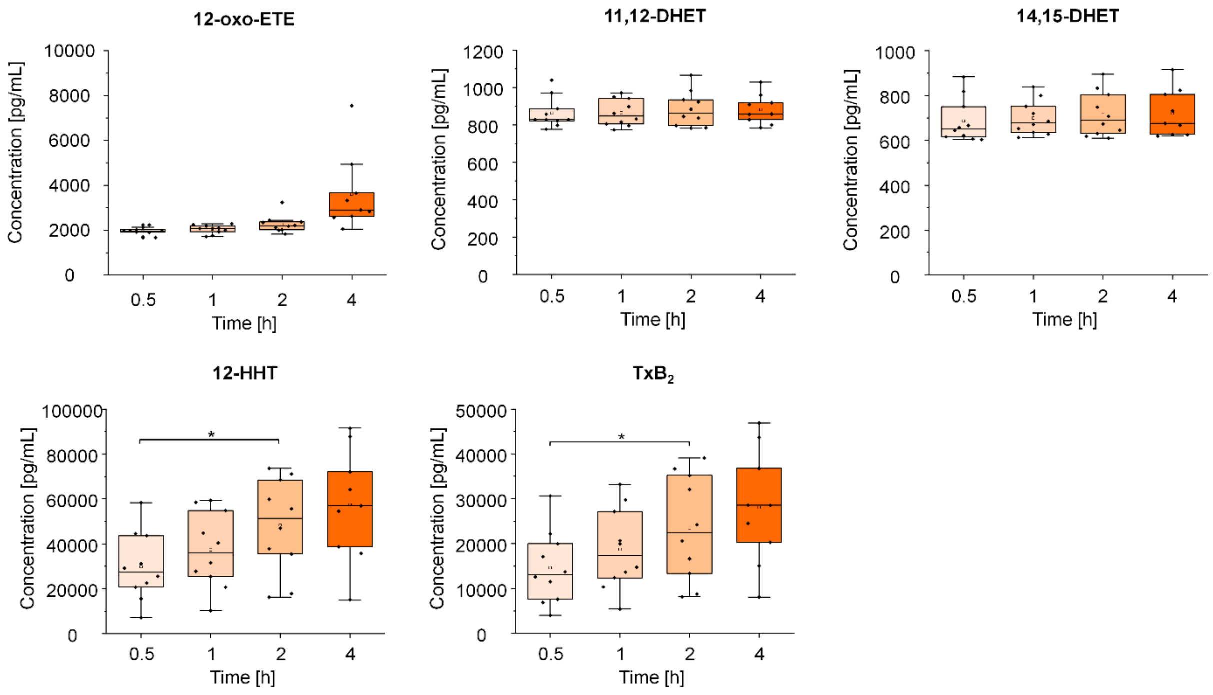

Figure S5: Influence of precentrifugation delay on eicosanoids in serum

Boxplots visualize the variation of the concentration of selected eicosanoids in human serum of healthy volunteers after prolonged incubation for 0.5, 1, 2 and 4 h at room temperature (*test cohort 1*, n=10) before centrifugation. Asterisks indicate differences between the time points as indicated by the respective brackets (*p<0.05). P-Values were calculated using linear mixed models or paired *t*-tests and corrected for multiple testing using Bonferroni post-hoc test.

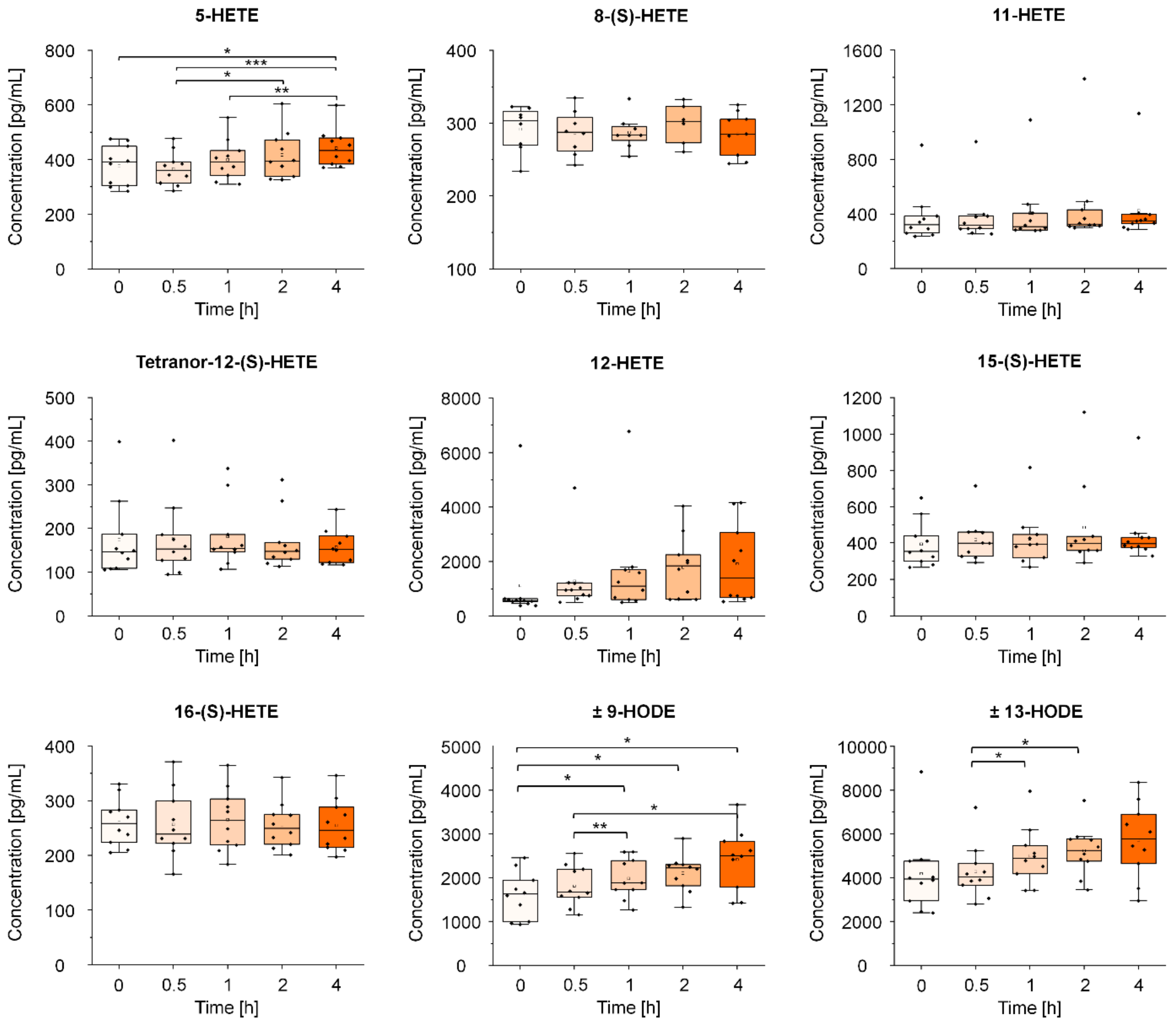

Figure S6A: Influcence of precentrifugation delay on eicosanoids in EDTA plasma

Boxplots visualize the variation of the concentration of selected eicosanoids in human EDTA plasma of healthy volunteers after prolonged incubation for 0, 0.5, 1, 2 and 4 h at room temperature (*validation sample 1*, n=10) before centrifugation. Asterisks indicate differences between the time points as indicated by the respective brackets (*p<0.05, **p<0.01, ***p<0.001). P-Values were calculated using linear mixed models or paired *t*-tests and corrected for multiple testing using Bonferroni post-hoc test.

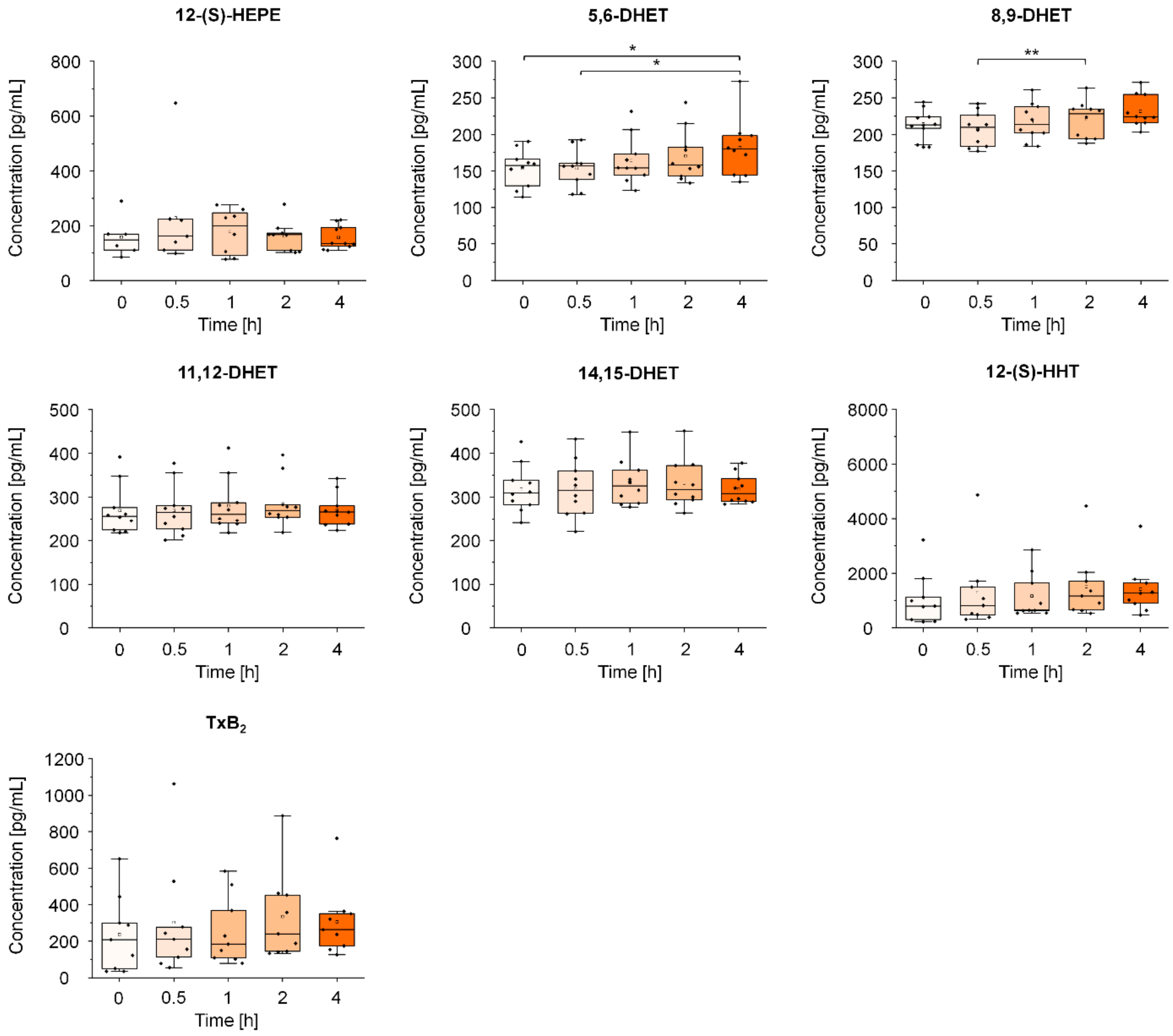

Figure S6B: Influcence of precentrifugation delay on eicosanoids in EDTA plasma

Boxplots visualize the variation of the concentration of selected eicosanoids in human EDTA plasma of healthy volunteers after prolonged incubation for 0, 0.5, 1, 2 and 4 h at room temperature (*validation sample 1*, n=10) before centrifugation. Asterisks indicate differences between the time points as indicated by the respective brackets (*p<0.05, **p<0.01). P-Values were calculated using linear mixed models or paired *t*-tests and corrected for multiple testing using Bonferroni post-hoc test.

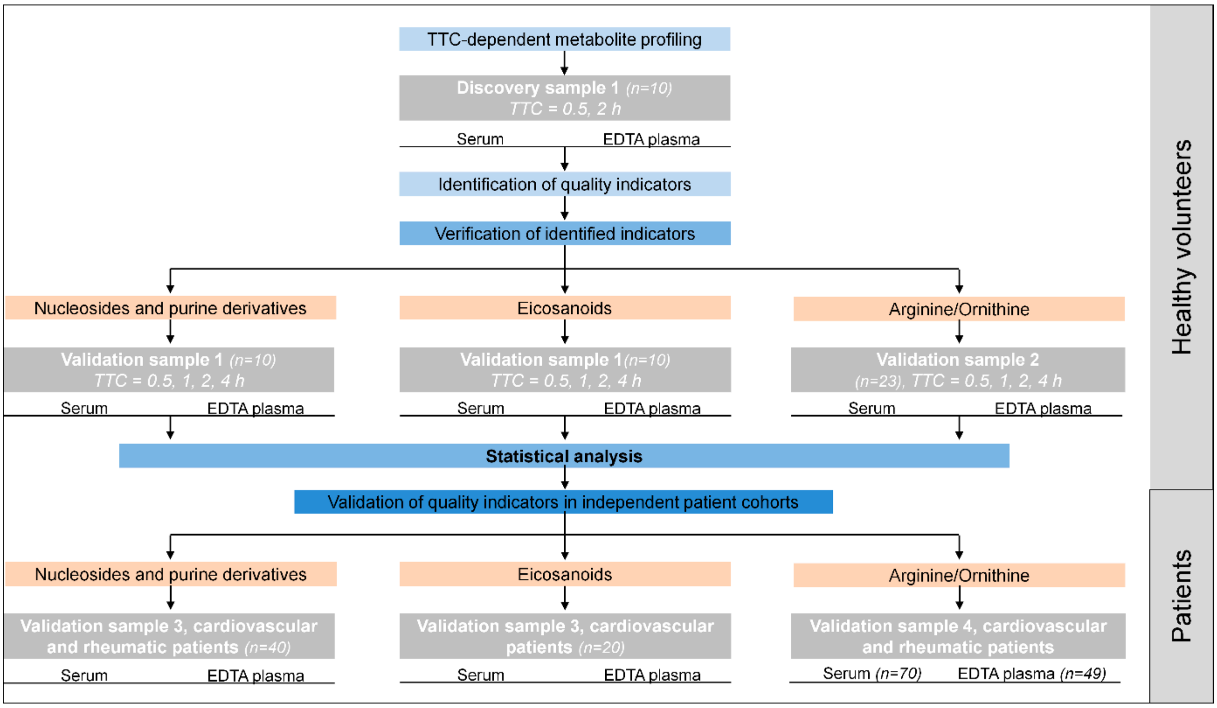

Figure S7: Experimental Design

Schematic overview of the QI study design describing the *discovery* and *validation* (1-4) *samples* as well as analytical parameters measured.
